# Evaluation of the relationship between thrombus burden, inflammation indices, and prognosis in central and peripheral pulmonary embolism

**DOI:** 10.1186/s12959-026-00873-6

**Published:** 2026-05-13

**Authors:** Bengü Kaan, Şeyma Başlılar, Ayşe Çapar, Cemre Abacı, Yahya Baraç

**Affiliations:** 1 Chest Diseases, Süreyyapaşa Chest Diseases and Thoracic Surgery, Training and Research Hospital, Istanbul, Turkey; 2 Chest Diseases, Sultan Abdulhamid Han Training and Research Hospital, Istanbul, Turkey; 3 Department of Anaesthesiology and Intensive Care Medicine, Sultan Abdulhamid Han Training and Research Hospital, Istanbul, Turkey; 4 Department of Radiology, Sultan Abdulhamid Han Training and Research Hospital, Istanbul, Turkey

**Keywords:** Pulmonary embolism, Thrombus localization, Thrombus burden, Inflammation indices, Mortality

## Abstract

**Objective:**

Early risk stratification is critical to clinical management of acute pulmonary embolism (APE). We aimed to evaluate the relationships among thrombus localization, thrombus burden, and inflammatory indices in patients with APE, and to assess the prognostic value of these parameters.

**Materials and methods:**

This retrospective, single-center study included adult patients with computed tomography pulmonary angiography (CTPA)-confirmed APE. Patients with active infection, hematologic or active solid organ malignancy, or immunosuppressive therapy were excluded. The cohort comprised 821 patients. Thrombus localization was assessed by CTPA and classified as central or peripheral. Thrombus burden was quantified using the Qanadli score. The Systemic Immune-Inflammation Index (SII), Systemic Inflammatory Response Index (SIRI), and Aggregate Index of Systemic Inflammation (AISI) were calculated from hematologic parameters at admission. Clinical, laboratory, and echocardiographic data were analyzed to assess 28-day mortality.

**Results:**

In patients with central thrombus location, the Qanadli score, right heart overload findings, pulmonary artery pressure, troponin, and B-type natriuretic peptide (pro-BNP) levels were significantly higher (*p* < 0.05). However, no significant relationship was found between thrombus localization and 28-day mortality, and the Qanadli score was also not associated with mortality (*p* > 0.05). SII, SIRI, and AISI values were significantly higher among patients who died (*p* < 0.05). In multivariate analyses, age, pro-BNP, C-reactive protein (CRP), red cell distribution width (RDW), and SII were identified as independent predictors of mortality (*p* < 0.05). In the receiver operating characteristics (ROC) analysis, SII showed the highest discriminatory power for predicting mortality (AUC = 0.748; *p* < 0.001).

**Conclusion:**

In APE, although thrombus burden and localization may correlate with clinical severity, mortality appears to be primarily driven by systemic inflammation. SII can serve as a practical, complementary biomarker for early risk stratification.

## Introduction

Acute pulmonary embolism (APE) is the third most common cardiovascular disease after myocardial infarction and stroke, causing approximately 60,000–100,000 deaths per year in United States [1, 2]. However, an ideal diagnostic and predictive tool that reliably reflects disease severity and prognosis has not yet been clearly defined. One of the most commonly used risk classification tools in clinical practice is the Pulmonary Embolism Severity Index (PESI), which primarily assesses patients’ clinical characteristics and cardiac biomarkers but lacks information about the degree and location of pulmonary arterial obstruction [3, 4].

There are a few studies focusing on this issue. The Qanadli score was developed to evaluate thrombus load based on the degree of occlusion in the pulmonary arterial system on computed tomography pulmonary angiography (CTPA) [4, 5]. However, the literature reports inconsistent and conflicting results on the relationship between this score and the clinical severity and prognosis of APE, with some studies demonstrating an association with clinical severity, while others report limited prognostic value for mortality [5, 6]. It is generally accepted that emboli in the main pulmonary artery are associated with more severe clinical outcomes [7], whereas peripheral thrombi have more limited hemodynamic effects [8].

APE is considered a complex thrombo-inflammatory condition in which a strong bidirectional interaction between thrombosis and inflammation has been demonstrated [9]. In this context, endothelial injury, platelet activation, and leukocyte recruitment contribute not only to thrombus formation but also to pulmonary vascular dysfunction, hypoxia, and a systemic inflammatory response, which may ultimately influence clinical outcomes [8]. Based on this pathophysiological approach, inflammatory indices have increasingly been used in the literature to predict prognosis and guide treatment strategies in pulmonary embolism (PE). The Systemic Immune-Inflammation Index (SII), the Systemic Inflammatory Response Index (SIRI), and the Aggregate Index of Systemic Inflammation (AISI) are composite biomarkers that reflect both the immune response and the inflammatory state. Although all three indices have been linked to adverse outcomes in APE, their relative and complementary prognostic value remains unclear [3, 10, 11].

This study aimed to evaluate relationships among thrombus location (central versus peripheral), thrombus burden, and blood count-based inflammatory indices (SII, SIRI, and AISI) in APE.

## Materials and methods

### Study design and patient population

This is a retrospective, single-center, observational study conducted at the Chest Diseases Clinic and the Chest Diseases Intensive Care Unit (ICU) of the Sultan Abdülhamid Han Training and Research Hospital. Patients aged 18 years or older who presented to our hospital between January 1, 2016, and June 30, 2025, with a confirmed diagnosis of APE by CTPA were included in the study. Patients with available clinical, laboratory, and imaging data were included in the analysis. Patients with active hematologic malignancy or hematologic disease, active infection, immunosuppressive therapy (high-dose steroids or immunomodulatory agents), or active solid malignancy undergoing chemotherapy or radiotherapy were excluded from the study.

### Data collection

Patients’ demographic characteristics (age, gender), clinical findings at presentation (dyspnea, chest pain, hemoptysis, syncope), comorbidities (hypertension (HT), diabetes mellitus (DM), chronic obstructive pulmonary disease (COPD), atrial fibrillation (AF), malignancy, deep vein thrombosis (DVT), and smoking history were recorded via the hospital information management system. Vital signs at admission included systolic blood pressure (SBP), diastolic blood pressure (DBP), and heart rate (HR). Laboratory data included: complete blood count parameters (hemoglobin, lymphocytes, neutrophils, monocytes, platelets, and red cell distribution width (RDW)); C-reactive protein (CRP); D-dimer; troponin; B-type natriuretic peptide (pro-BNP); albumin levels; and arterial blood gas parameters (pH, peripheral oxygen saturation (SPO_2_)).

Echocardiographic evaluations recorded right ventricular overload findings and pulmonary artery pressure (PAP). At the widest point of the ventricle, right ventricular diameter/left ventricular diameter > 0.9 and interventricular septal deviation were considered right ventricular overload findings [12]. Additionally, the need for ICU, the duration of hospital stay, and whether mortality occurred within 28 days were noted. Furthermore, patient risk classification was determined and recorded in accordance with the 2019 European Society of Cardiology (ESC) guidelines [13].

### Calculation of inflammation indices

The following inflammation indices were calculated using complete blood count parameters:

- **SII**: Platelets × Neutrophils / Lymphocytes
- **SIRI**: Neutrophils × Monocyte / Lymphocyte
- **AISI**: Neutrophil × Monocyte × Platelet / Lymphocyte

These indices have previously been evaluated as prognostic markers in APE and were calculated using widely used definitions in the literature; however, their values may be influenced by underlying comorbid conditions [3, 10] .

### Imaging and thrombus assessment

The location and burden of thrombus associated with PE were determined by evaluating CTPA images. Thrombus localization was classified as central (pulmonary trunk and/or main pulmonary artery/arteries) or peripheral (lobar/segmental/subsegmental branches) [8]. Thrombus burden was quantified using the Qanadli score. The Qanadli score is a validated method for quantifying the extent of arterial obstruction and is based on the degree of embolism occlusion in 20 pulmonary segmental arteries. Each pulmonary artery receives 2 points for complete embolism, 1 point for partial embolism, and 0 points for no embolism, allowing the obstruction score to reach a maximum of 40 points. This scoring system has been widely validated in patients with APE and has been shown to correlate with clinical severity [4]. All imaging evaluations were performed by a single experienced radiologist.

### End points

Primary endpoint: Evaluation of the relationship among the thrombus location, thrombus burden, and inflammatory indices (SII, SIRI, and AISI).

Secondary endpoint: Relationship of these parameters with prognosis and mortality.

### Statistical analysis

Statistical analyses were performed using IBM Statistical Packages for the Social Sciences (SPSS) Statistics 30. Analyses were conducted using available data for each variable, and cases with missing values were excluded from the corresponding analyses. No imputation methods were used. The distribution of continuous variables was assessed, and data were presented as mean ± SD or median (IQR), as appropriate. Categorical variables were expressed as numbers and percentages. For comparisons between the central and peripheral thrombosis groups, the independent-samples t-test or the Mann–Whitney U test was used for continuous variables, as appropriate. Categorical variables were analyzed using the chi-square test or Fisher’s exact test, as appropriate. For 28-day mortality, univariate and multivariate analyses were performed using Cox regression in survival analysis to identify independent predictors. To minimize confounding, variables included in the multivariate model were selected based on univariate analysis results and clinical relevance. Additionally, to avoid overfitting, the number of variables in the model was limited according to the number of events. To avoid multicollinearity, variables representing similar biological processes were not included together in the multivariate model. The performance of inflammatory indices in predicting mortality was evaluated using receiver operating characteristic (ROC) curve analysis; area under the curve (AUC), cutoff, sensitivity, specificity, and Youden index were calculated. Kaplan–Meier survival curves were plotted for groups defined by the ROC-derived cutoff, and the groups were compared using the log-rank test. The level of statistical significance was set at *p* < 0.05.

### Ethical approval

The study protocol received approval from the Non-Interventional Clinical Research Ethics Committee at Health Science University Umraniye Training and Research Hospital (B.10.1.TKH.4.34.H.GP.0.01/452). Because of the retrospective design, informed consent was waived. The study was conducted in accordance with the principles outlined in the Declaration of Helsinki.

## Results

A total of 821 patients with a median age of 72.00 years (IQR: 59.00–00) were included. Of these, 364 (44.3%) were male, and 457 (55.7%) were female, 50.7% had HT, 22.8% had DM, 11.4% had COPD, 13% had AF, and 20.4% had malignancy. The most common presenting symptom was dyspnea (68.2%), followed by chest pain (31.2%), hemoptysis (9.3%), and syncope (8.5%). 36.3% were smokers. The thrombus was centrally located in 48.4% of patients (*n* = 397) and peripherally located in 51.6% (*n* = 424). The median Qanadli score was 10.00 (IQR: 5.00–20.00). According to the ESC risk classification, 41.1% of patients were low risk, 34.3% were low-to-moderate risk, 18.5% were moderate-to-high risk, and 6.2% were high risk. The 28-day mortality rate was 5.3% (*n* = 43). Right ventricular overload was revealed in 24.7%, and the median PAP was 25.00 (IQR: 20.00–40.00). 28.7% of patients had DVT. The median vital signs were as follows: SBP 120.00 (IQR: 113.00-135.00), DBP 80.00 (IQR: 70.00–81.00), and HR 95.00 (IQR: 82.00-103.50). 25.2% of patients required ICU, and the median hospital stay was 7.00 (IQR: 5.00–10.00) (Table 1).

**Table 1:** Baseline demographic, clinical, laboratory, and radiological characteristics of patients with pulmonary embolism

Variables	Mean±SD, Median (Q1-Q3) or *n* (%)
**Gender**	
Male Female	364 (44.3) 457 (55.7)
Age	72.00 (59.00–84.00)
Dyspnea	560 (68.2)
Chest Pain	256 (31.2)
Hemoptysis	76 (9.3)
Syncope	70 (8.5)
Smoking	298 (36.3)
SBP mm-Hg	120.00 (113.00-135.00)
DBP mm-Hg	80.00 (70.00–81.00)
HR bpm	95.00 (82.00-103.50)
Hemoglobin g/dl	12.06±2.21
Lymphocyte⋅ 10^9^/L	1.61 (1.07–2.21)
Neutrophil⋅ 10^9^/L	6.49 (4.63–9.41)
Monocyte⋅ 10^9^/L	0.56 (0.41–0.76)
Platelet⋅ 10^9^/L	234.00 (184.00-298.00)
D-dimer mg/L	3.60 (1.80-4.00)
Albumin g/L	36.00 (31.00–40.00)
Troponin ng/L	19.00 (6.92-53.00)
pro-BNP pg/ml	299.00 (64.75-1498.50)
pH	7.43 (7.38–7.45)
SPO_2_%	92.00 (87.00–95.00)
RDW %	14.30 (13.20–15.80)
CRP mg/L	41.00 (14.00–99.00)
DVT	219 (28.7)
PAP mm-Hg	25.00 (20.00–40.00)
RV strain	200 (24.7)
**Risk stratification**	
Low risk Intermediate-low risk Intermediate-high risk High risk	333 (41.1) 278 (34.3) 150 (18.5) 50 (6.2)
28-day mortality	43 (5.3)
ICU need	207 (25.2)
Length of hospital stay (days)	7.00 (5.00–10.00)
Thrombolytic therapy	60 (7.4)
HT	416 (50.7)
DM	187 (22.8)
COPD	93 (11.4)
AF	107 (13.0)
Malignancy	167 (20.4)
SII	984.69 (555.69-1708.50)
SIRI	2.26 (1.22–4.27)
AISI	512.56 (268.21-1055.54)
**Thrombus location**	
Central Peripheral	397 (48.4) 424 (51.6)
Qanadli score	10.00 (5.00–20.00)

SBP: Systolic Blood Pressure, DBP: Diastolic Blood Pressure, HR: Heart Rate, BNP: Brain Natriuretic Peptide, SPO2: Peripheral Capiller Oxygen Saturation, RDW: Red Cell Distribution Width, CRP: C-Reactive Protein, DVT: Deep Venous Thrombosis, PAP: Pulmonary Artery Pressure, RV: Right Ventricle, ICU: Intensive Care Unit, HT: Hypertension, DM: Diabetes Mellitus, COPD: Chronic Obstructive Pulmonary Diseases, AF: Atrial Fibrillation, SII: Systemic Immune-Inflammation Index, SIRI: Systemic Inflammation Response Index, AISI: Aggregate Index of Systemic Inflammation

Laboratory findings: hemoglobin 12.06 ± 2.21, lymphocyte 1.61 (IQR: 1.07–2.21), neutrophil 6.49 (IQR: 4.63–9.41), monocyte 0.56 (IQR: 0.41–0.76), platelets 234.00 (IQR: 184.00-298.00), D-dimer 3.60 (IQR: 1.80-4.00), albumin 36.00 (IQR: 31.00–40.00), troponin 19.00 (IQR: 6.92-53.00), and pro-BNP 299.00 (IQR: 64.75-1498.50). Median inflammatory indices were as follows: SII 984.69 (IQR: 555.69-1708.50), SIRI 2.26 (IQR: 1.22–4.27), and AISI 512.56 (IQR: 268.21–1055) (Table 1).

### Clinical and laboratory findings based on thrombus location

In the central thrombus group, hemoptysis was less common, whereas syncope was more frequent (6.8% vs. 11.6%; *p* = 0.019 and 12.8% vs. 4.5%; *p* < 0.001, respectively). Although cigarette use was lower in the central group (30.9% vs. 43.3%; *p* < 0.001), the rate of concomitant DVT was higher (35.1% vs. 22.7%; *p* < 0.001) (Table 2).

**Table 2:** Clinical and laboratory characteristics according to thrombus location in pulmonary embolism

Variables	Central pulmonary embolism Mean±SD, Median (Q1-Q3) or *n* (%) *n* = 397	Peripheral pulmonary embolism Mean±SD, Median (Q1-Q3) or *n* (%) *n* = 424	*p*-value
**Gender**			
Female Male	232 (58.4) 165 (41.6)	225 (53.1) 199 (46.9)	0.122
Age	73.00 (62.00–84.00)	73.00 (60.00–85.00)	0.102
Dyspnea	274 (69.0)	286 (67.5)	0.630
Chest Pain	122 (30.7)	134 (31.6)	0.787
Hemoptysis	27 (6.8)	49 (11.6)	**0.019**
Syncope	51 (12.8)	19 (4.5)	**< 0.001**
Smoking	118 (30.9)	180 (43.3)	**< 0.001**
SBP mm-Hg	120.00 (114.00-140.00)	120.00 (116.00-130.00)	0.303
DBP mm-Hg	80.00 (70.00–80.00)	80.00 (70.00–80.00)	0.325
HR bpm	95.00 (82.00-104.00)	95.00 (82.00-102.00)	0.349
Hemoglobin g/dl	12.08±2.13	11.99±2.18	0.828
Lymphocyte ⋅ 10^9^/L	1.63 (1.09–2.25)	1.62 (1.06–2.16)	0.373
Neutrophil ⋅ 10^9^/L	6.95 (5.08–9.24)	6.00 (4.21–9.24)	**0.004**
Monocyte ⋅ 10^9^/L	0.58 (0.41–0.76)	0.56 (0.41–0.77)	0.623
Platelet ⋅ 10^9^/L	225.00 (179.00-276.00)	237.00 (190.25–310.00)	**0.007**
D-dimer mg/L	4.00 (2.24-4.00)	2.93 (1.40-4.00)	**< 0.001**
Albumin g/L	36.00 (32.00–40.00)	35.00 (31.00–40.00)	0.841
Troponin ng/L	28.00 (9.00–72.00)	13.00 (5.00-31.75)	**< 0.001**
pro-BNP pg/ml	353.00 (93.00-1347.00)	184.00 (50.00-1063.25)	**< 0.001**
pH	7.43 (7.39–7.45)	7.43 (7.38–7.45)	**0.020**
SPO_2_%	91.00 (86.00–95.00)	93.00 (88.00–95.00)	**0.002**
RDW %	14.20 (13.20–15.80)	14.40 (13.30-15.98)	0.900
CRP mg/L	42.00 (18.00–90.00)	40.00 (12.93–99.75)	0.644
DVT	130 (35.1)	89 (22.7)	**< 0.001**
PAP mm-Hg	30.00 (20.00–45.00)	25.00 (20.00–35.00)	**< 0.001**
RV strain	135 (34.4)	65 (15.6)	**< 0.001**
**Risk stratification**			
Low risk Intermediate-low risk Intermediate-high risk High risk	118 (30) 137 (34.9) 104 (26.5) 34 (8.7)	215 (51.4) 141 (33.7) 46 (11) 16 (3.8)	**< 0.001**
28-day mortality	21 (5.3)	22 (5.2)	0.967
ICU need	129 (32.5)	78 (18.4)	**< 0.001**
Length of hospital stay (days)	7.00 (5.00–11.00)	7.00 (5.00–10.00)	**0.006**
Thrombolytic therapy	50 (12.6)	10 (2.4)	**< 0.001**
HT	203 (51.1)	213 (50.4)	0.824
DM	98 (24.7)	89 (21.0)	0.214
KOAH	30 (7.6)	63 (14.9)	**< 0.001**
AF	52 (13.1)	55 (13.0)	0.968
Malignancy	77 (19.4)	90 (21.3)	0.504
SII	939.58 (565.98-1624.80)	970.39 (524.16-1926.25)	0.861
SIRI	2.49 (1.34–4.27)	2.02 (1.18–4.10)	0.169
AISI	523.00 (286.77-1025.37)	486.16 (255.69-1087.46)	0.840
Qanadli score	18.00 (10.00–21.00)	6.00 (2.25-10.00)	**< 0.001**

SBP: Systolic Blood Pressure, DBP: Diastolic Blood Pressure, HR: Heart Rate, BNP: Brain Natriuretic Peptide, SPO2: Peripheral Capiller Oxygen Saturation, RDW: Red Cell Distribution Width, CRP: C-Reactive Protein, DVT: Deep Venous Thrombosis, PAP: Pulmonary Artery Pressure, RV: Right Ventricle, ICU: Intensive Care Unit, HT: Hypertension, DM: Diabetes Mellitus, COPD: Chronic Obstructive Pulmonary Diseases, AF: Atrial Fibrillation, SII: Systemic Immune-Inflammation Index, SIRI: Systemic Inflammation Response Index, AISI: Aggregate Index of Systemic Inflammation. Independent samples t-test or Mann–Whitney U test was used for continuous variables, as appropriate. Categorical variables were compared using the chi-square test or Fisher’s exact test

In laboratory evaluations, the neutrophil count in patients with central thrombosis (6.95 [IQR: 5.08–9.24] vs. 6.00 [IQR: 4.21–9.24], *p* = 0.004), D-dimer (4.00 [IQR: 2.24-4.00] vs. 2.93 [IQR: 1.40-4.00], *p* < 0.001), troponin (28.00 [IQR: 9.00–72. 00] vs. 13.00 [IQR: 5.00-31.75], *p* < 0.001) and pro-BNP (353.00 [IQR: 93.00-1347. 00] vs. 184.00 [IQR: 50.00-1063.25], *p* < 0.001) levels were significantly higher. Platelet count was lower in the central embolism group (225.00 [IQR: 179.00-276.00] vs. 237.00 [IQR: 190.25–310.00], *p* = 0.007) (Table 2).

Additionally, in this group, SPO_2_ was lower (91.00 [IQR: 86.00–95.00] vs. 93.00 [IQR: 88.00–95.00], *p* = 0.002), PAP was higher (30.00 [IQR: 20.00–45.00] vs. 25.00 [IQR: 20.00–35.00], *p* < 0.001), and right ventricular overload was observed more frequently (34.4% vs. 15.6%, *p* < 0.001). The Qanadli score was significantly higher in the central group (18.00 [IQR: 10.00–21.00] vs. 6.00 [IQR: 2.25–10.00]; *p* < 0.001) (Table 2).

In accordance with the ESC risk classification, high-risk patients were more prevalent in the central embolism group (intermediate-high 26.5% vs. 11%, high 8.7% vs. 3.8%, *p* < 0.001). The need for ICU, hospital stay duration, and thrombolytic therapy were higher in the central embolism group (32.5% vs. 18.4%, *p* < 0.001; 7.00 [IQR:5.00–11.00] vs. 7.00 [IQR:5.00–10.00], *p* = 0.006; 12.6% vs. 2.4%, *p* < 0.001; respectively) (Table 2).

**Fig. 1:**
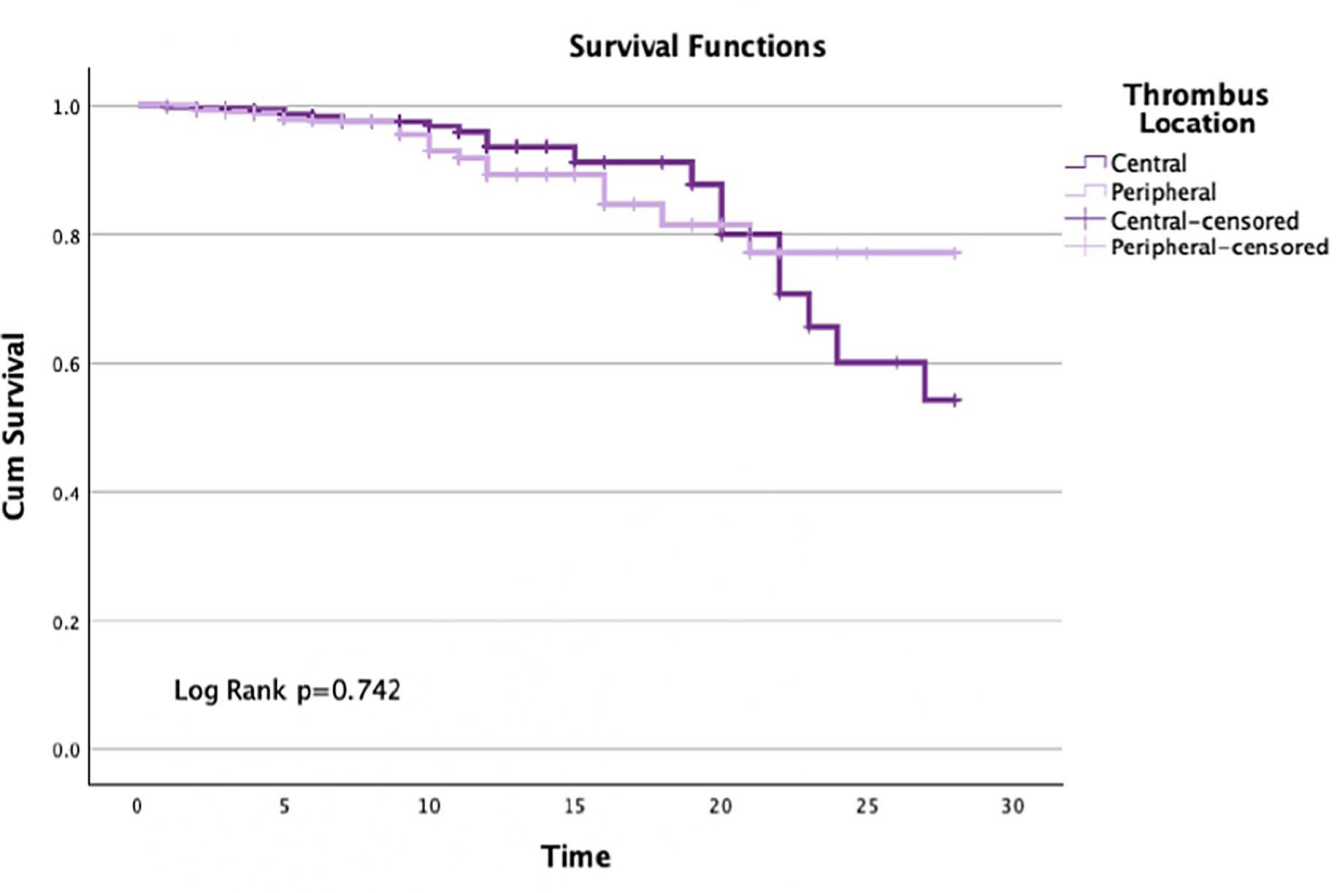
Kaplan-Meier survival curves for 28-day mortality comparing central and peripheral PE. No significant difference was observed between groups (log-rank *p* = 0.742). PE: Pulmonary Embolism

On the other hand, SII, SIRI, and AISI values did not differ significantly by thrombus location (939.58 [IQR: 565.98-1624.80] vs. 970.39 [IQR: 524-1926.25], *p* = 0.861; 2.49 [IQR: 1.34–4.27] vs. 2.02 [IQR: 1.18–4.10], *p* = 0.169; 523.00 [IQR: 286.77-1025.37] vs. 486.16 [IQR: 255.69-1087.46], *p* = 0.840). The 28-day mortality rates were similar between the central and peripheral groups (5.3% vs. 5.2%, *p* = 0.967). No significant differences were observed between the groups in other laboratory and clinical findings (*p* > 0.05) (Table 2) (Fig. 1).

### 28-day mortality determinants

Patients who died within 28 days were older (85.00 [IQR: 75.00–89.00] vs. 72.00 [IQR: 60.00–84.00], *p* < 0.001), had more frequent dyspnea (83.7% vs. 67.4%, *p* = 0.025), had lower SPO_2_ (85.00 [IQR: 78.00–90.00] vs. 92.00 [IQR: 88.00–95.00], *p* < 0.001), had longer hospital stays, and had a higher need for ICU (10.00 [IQR: 6.00–16.00] vs. 7.00 [IQR: 5.00–10.00], *p* = 0.017; 86% vs. 21.8%, *p* < 0.001, respectively) (Table 3).

**Table 3:** Clinical and laboratory characteristics according to 28-day mortality in pulmonary embolism

Variables	Non-survivors at 28 days Median (Q1-Q3) or *n* (%)	Survivors at 28 days Median (Q1-Q3) or *n* (%)	*p*-value
**Gender**			0.979
Male Female	19 (44.2) 24 (55.8)	344 (44.4) 431 (55.6)	
Age	85.00 (75.00–89.00)	72.00 (60.00–84.00)	**< 0.001**
Dyspnea	36 (83.7)	522 (67.4)	**0.025**
Chest Pain	8 (18.6)	247 (31.9)	0.068
Hemoptysis	0 (0.0)	75 (9.7)	**0.032**
Syncope	7 (16.3)	61 (7.9)	0.052
Smoking	14 (35.0)	283 (37.5)	0.752
SBP mm-Hg	118.00 (100.00-130.00)	120.00 (116.00-135.00)	**0.031**
DBP mm-Hg	70.00 (60.00–82.00)	80.00 (70.00–80.00)	0.127
HR bpm	100.00 (88.00-120.00)	95.00 (82.00-102.00)	**0.002**
Hemoglobin g/dl	11.40 (10.20–12.50)	12.05 (10.40–13.60)	**0.032**
Lymphocyte ⋅ 10^9^/L	1.40 (0.76–1.88)	1.63 (1.09–2.26)	**0.007**
Neutrophil ⋅ 10^9^/L	10.82 (7.86–14.21)	6.37 (4.59–8.86)	**< 0.001**
Monocyte ⋅ 10^9^/L	0.59 (0.40–0.94)	0.57 (0.41–0.76)	0.552
Platelet ⋅ 10^9^/L	254.00 (172.00-377.00)	231.00 (184.00-292.75)	0.242
D-dimer mg/L	4.00 (2.00–4.00)	3.55 (1.80-4.00)	0.115
Albumin g/L	30.00 (25.00–34.00)	36.00 (32.00–40.00)	**< 0.001**
Troponin ng/L	60.00 (31.00-114.00)	17.50 (6.00-48.75)	**< 0.001**
pro-BNP pg/ml	1443.00 (627.00-8721.00)	240.50 (55.00-1093.75)	**< 0.001**
pH	7.41 (7.35–7.47)	7.43 (7.39–7.45)	0.154
SPO_2_%	85.00 (78.00–90.00)	92.00 (88.00–95.00)	**< 0.001**
RDW %	16.80 (15.00-20.50)	14.19 (13.20–15.70)	**< 0.001**
CRP mg/L	72.00 (48.00-130.00)	39.00 (14.00-92.75)	**< 0.001**
DVT	8 (24.2)	211 (29.0)	0.556
PAP mm-Hg	40.00 (25.00–50.00)	25.00 (20.00–40.00)	**0.006**
RV srain	21 (48.8)	178 (23.2)	**< 0.001**
**Risk Stratification**			**< 0.001**
Low risk Intermediate-low risk Intermediate-high risk High risk	1 (2.3) 19 (44.2) 13 (30.2) 10 (23.3)	332 (43.2) 259 (33.7) 137 (17.8) 40 (5.2)	
ICU need	37 (86.0)	169 (21.8)	**< 0.001**
Length of hospital stay (days)	10.00 (6.00–16.00)	7.00 (5.00–10.00)	**0.017**
Thrombolytic therapy	6 (14.3)	54 (7.0)	0.079
HT	25 (58.1)	391 (50.5)	0.326
DM	12 (27.9)	175 (22.6)	0.418
KOAH	7 (16.3)	86 (11.1)	0.299
AF	12 (27.9)	94 (12.1)	**0.003**
Malignancy	12 (27.9)	153 (19.7)	0.194
SII	2246.16 (1473.57-3851.76)	936.94 (536.59-1605.50)	**< 0.001**
SIRI	4.97 (2.41–9.10)	2.20 (1.22–3.99)	**< 0.001**
AISI	1199.02 (596.79-2728.08)	485.58 (266.24-1009.88)	**< 0.001**
**Thrombus location**			0.967
Central Peripheral	21 (48.8) 22 (51.2)	376 (48.5) 399 (51.5)	
Qanadli score	9.00 (4.00–16.00)	10.00 (5.00–20.00)	0.219

SBP: Systolic Blood Pressure, DBP: Diastolic Blood Pressure, HR: Heart Rate, BNP: Brain Natriuretic Peptide, SPO2: Peripheral Capiller Oxygen Saturation, RDW: Red Cell Distribution Width, CRP: C-Reactive Protein, DVT: Deep Venous Thrombosis, PAP: Pulmonary Artery Pressure, RV: Right Ventricle, ICU: Intensive Care Unit, HT: Hypertension, DM: Diabetes Mellitus, COPD: Chronic Obstructive Pulmonary Diseases, AF: Atrial Fibrillation, SII: Systemic Immune-Inflammation Index, SIRI: Systemic Inflammation Response Index, AISI: Aggregate Index of Systemic Inflammation. Mann–Whitney U test was used for continuous variables. Categorical variables were compared using the chi-square test or Fisher’s exact test

In these patients, neutrophil count, troponin, pro-BNP, RDW, and CRP levels were significantly higher (10.82 [IQR: 7.86–14.21] vs. 6.37 [IQR: 4.59–8.86], *p* < 0.001; 60.00 [IQR: 31.00-114.00] vs. 17.50 [IQR: 6.00-48.75], *p* < 0.001; 1443.00 [IQR: 627.00-8721.00] vs. 240.50 [IQR: 55.00-1093.75], *p* < 0.001; 16.80 [IQR: 15.00-20.50] vs. 14.19 [IQR: 13.20–15.70], *p* < 0.001; 72.00 [IQR: 48.00-130. 00] vs. 39.00 [IQR: 14.00-92.75], *p* < 0.001; respectively). Hemoglobin, lymphocyte, albumin, and SPO_2_ levels were lower (11.40 [IQR: 10.20–12.50] vs. 12.05 [IQR: 10.40–13.60], *p* = 0.032; 1.40 [IQR: 0.76–1.88] vs. 1.63 [IQR: 1.09–2.26], *p* = 0.007; 30.00 [IQR: 25.00–34.00] vs. 36.00 [IQR: 32.00–40.00], *p* < 0.001; 85.00 [IQR: 78.00–90.00] vs. 92.00 [IQR: 88.00–95.00], *p* < 0.001; respectively) (Table 3).

The patients who died were in the intermediate-low, intermediate-high, and high-risk groups (44.2% (*n* = 19), 30.2% (*n* = 13), and 23.3% (*n* = 10) among non-survivors; 33.7% (*n* = 259), 17.8% (*n* = 137), and 5.2% (*n* = 40) among survivors, respectively) and had higher PAP values and more frequently had right ventricular dysfunction (40.00 [IQR: 25.00–50.00] vs. 25.00 [IQR: 20.00–40.00], *p* = 0.006; 48.8% vs. 23.2%, *p* < 0.001, respectively) (Table 3).

Inflammation indices were significantly higher in patients who died: SII: 2246.16 vs. 936.94, *p* < 0.001; SIRI: 4.97 vs. 2.20, *p* < 0.001; and AISI: 1199.02 vs. 485.58, *p* < 0.001. No significant association was found between thrombus location and the Qanadli score with 28-day mortality (central: 48.8% vs. 48.5%, peripheral: 51.2% vs. 51.5%, *p* = 0.967; 9.00 (IQR: 4.00–16.00) vs. 10.00 (IQR: 5.00–20.00), *p* = 0.219, respectively). There was no difference in mortality among patients with respect to other laboratory and clinical findings (*p* < 0.05) (Table 3).

### Regression and ROC analyses

In univariate regression analysis, age, neutrophil count, albumin, pro-BNP, RDW, CRP, SII, SIRI, and AISI were significantly associated with mortality (HR 1.038, *p* = 0.002; HR 1.139, *p* < 0.001; HR 0.938, *p* = 0.011; HR 1.000, *p* = 0.002; HR 1.249, *p* < 0.001; HR 1.004, *p* = 0.006; HR 1.000, *p* < 0.001; HR 1.059, *p* = 0.004; HR 1.000, *p* < 0.001; respectively), whereas in multivariate Cox regression analysis, age, pro-BNP, RDW, CRP, and SII were identified as independent predictors of 28-day mortality (HR 1.039, *p* = 0.005; HR 1.000, *p* = 0.036; HR 1.242, *p* < 0.001; HR 1.004, *p* = 0.033; HR 1.000, *p* < 0.001, respectively) (Table 4).

**Table 4:** Univariate and multivariate Cox regression analyses for 28-day mortality in patients with pulmonary embolism

	Univariate			Multivariate		
	HR %95 CI	B coefficient	*p*-value	HR %95 CI	B coefficient	*p*-value
Age	1.038 (1.014–1.062)	0.037	**0.002**	1.039 (1.011–1.067)	0.038	**0.005**
Hemoglobin g/dl	0.900 (0.785–1.031)	-0.106	0.128			
Lymphocyte⋅10^9^/L	0.710 (0.479–1.054)	-0.342	0.089			
Neutrophil⋅10^9^/L	1.139 (1.074–1.207)	0.130	**< 0.001**			
Albumin g/L	0.938 (0.893–0.985)	-0.064	**0.011**			
Troponin ng/L	1.000 (1.000-1.001)	0.000	0.557			
pro-BNP pg/ml	1.000 (1.000–1.000)	0.000	**0.002**	1.000 (1.000–1.000)	0.000	**0.036**
RDW %	1.249 (1.160–1.346)	0.223	**< 0.001**	1.242 (1.138–1.356)	0.217	**< 0.001**
CRP mg/L	1.004 (1.001–1.007)	0.004	**0.006**	1.004 (1.000-1.007)	0.004	**0.033**
AF	1.821 (0.928–3.572)	0.559	0.081			
SII	1.000 (1.000–1.000)	0.000	**< 0.001**	1.000 (1.000–1.000)	0.000	**< 0.001**
SIRI	1.059 (1.018–1.101)	0.057	**0.004**			
AISI	1.000 (1.000–1.000)	0.000	**< 0.001**			

BNP: Brain Natriuretic Peptide, RDW: Red Cell Distribution Width, CRP: C-Reactive Protein, AF: Atrial Fibrillation, SII: Systemic Immune-Inflammation Index, SIRI: Systemic Inflammation Response Index, AISI: Aggregate Index of Systemic Inflammation

**Fig. 2:**
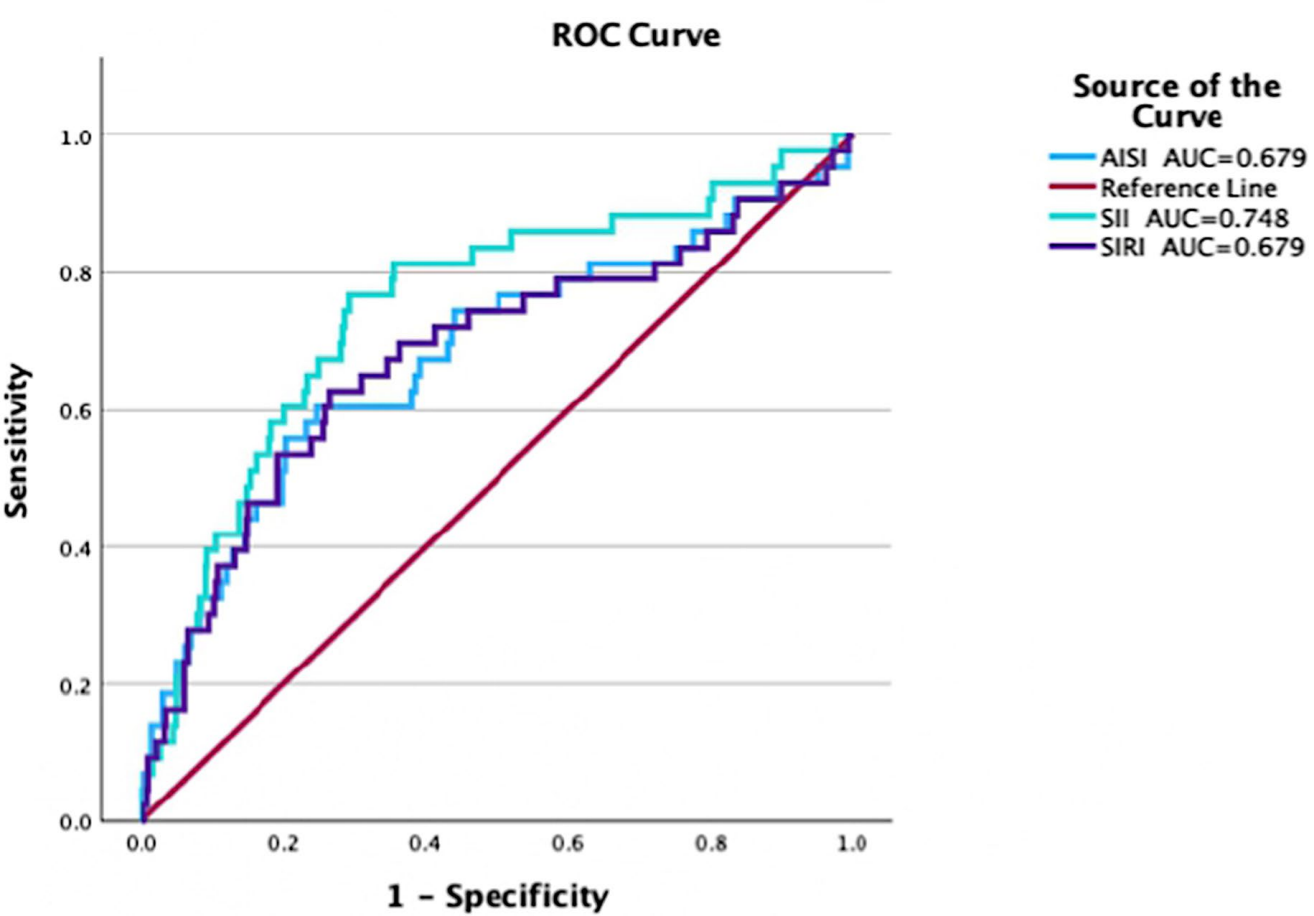
ROC curves showing the discriminative performance of systemic inflammatory indices (SII, SIRI, and AISI) for predicting 28-day mortality. SII: Systemic Immune-Inflammation Index, SIRI: Systemic Inflammation Response Index, AISI: Aggregate Index of Systemic Inflammation

**Fig. 3:**
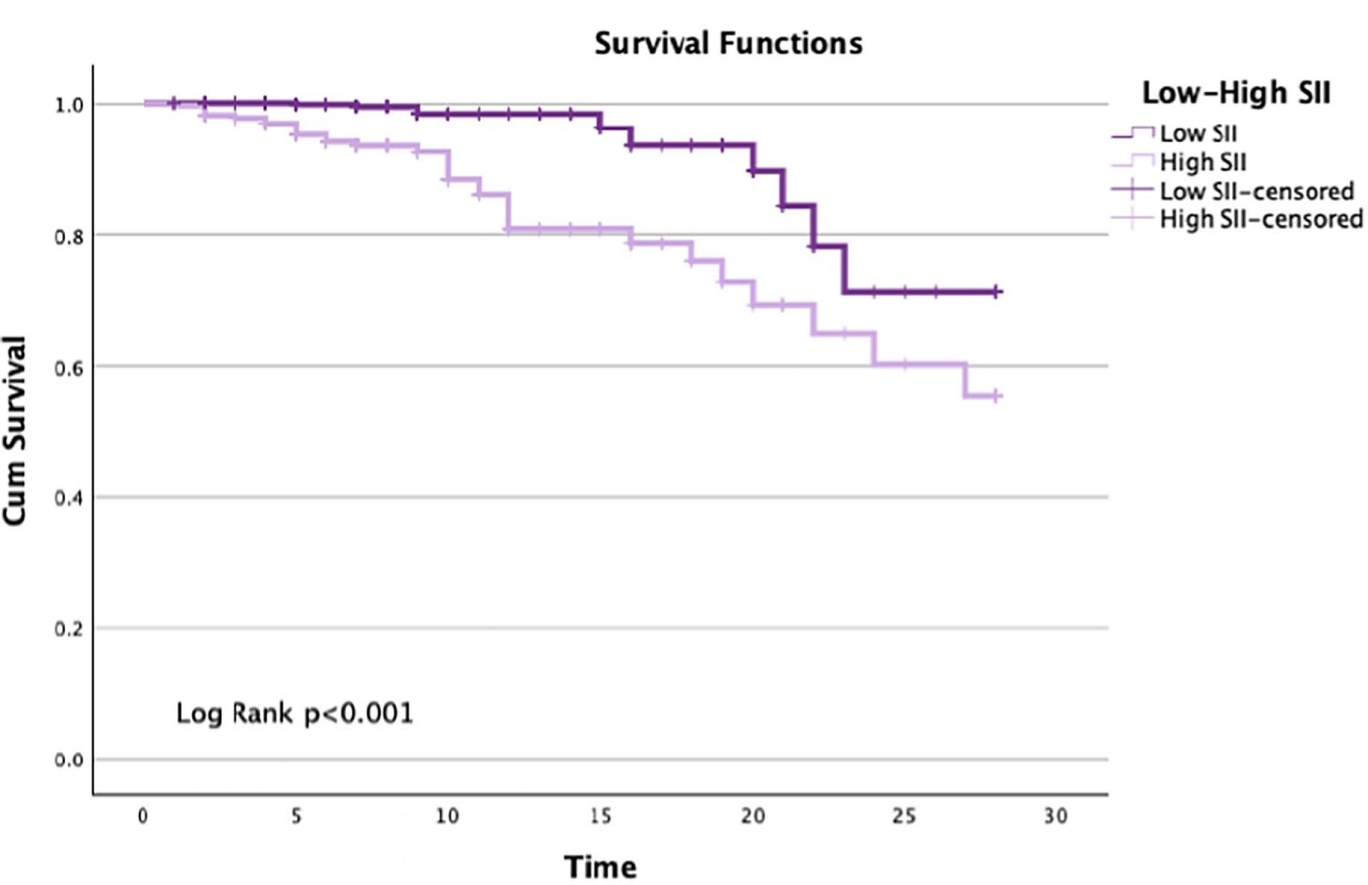
Kaplan–Meier survival curves for 28-day survival according to low and high SII groups, stratified using the ROC-derived cut-off value of 1472.89. Survival curves were compared using the log-rank test. SII: Systemic Immune-Inflammation Index

In ROC analyses, inflammation indices showed significant performance in predicting mortality: SII, AUC 0.748 (95% CI: 0.666–0.829); SIRI, AUC 0.679 (95% CI: 0.584–0.774); AISI, AUC 0.679 (95% CI: 0.584–0.774) (Fig. 2). Among these indices, SII was the most discriminative. Kaplan–Meier curves generated using the cut-off value of 1472.89, determined by ROC analysis, showed that 28-day survival was significantly lower in the high SII group (log-rank *p* < 0.001) (Table 5) (Fig. 3).

**Table 5:** ROC analysis of inflammatory indices for predicting 28-day mortality in patients with pulmonary embolism

Variables	AUC (95% CI)	Cut off value	Sensitivity	Specifity	Youden Index	*p*-value
SII	0.748 (0.666–0.829)	1472.89	0.767	0.708	0.476	**< 0.001**
SIRI	0.679 (0.584–0.774)	3.83	0.628	0.735	0.363	**< 0.001**
AISI	0.679 (0.584–0.774)	1021.54	0.605	0.754	0.358	**< 0.001**

SII: Systemic Immune-Inflammation Index, SIRI: Systemic Inflammation Response Index, AISI: Aggregate Index of Systemic Inflammation

The DeLong test showed that the AUC for SII was significantly higher than that for AISI (*p* = 0.044). However, no statistically significant differences were observed between SII and SIRI (*p* = 0.068) or between SIRI and AISI (*p* = 0.991).

## Discussion

In this study, the relationships among thrombus localization, thrombus load, and hemogram-based inflammatory indices were evaluated in a large cohort of patients diagnosed with APE. Thus, the effects of both the thrombotic and inflammatory components of the disease on clinical severity and outcomes were investigated. Our findings demonstrate that inflammatory indices are strongly associated with 28-day mortality, independent of thrombus location. In contrast, central thrombus location is associated with greater thrombus burden, higher Qanadli score, marked right heart overload, and increased clinical severity.

In this study, patients with central PE had higher Qanadli scores, which may reflect thrombus extending from the proximal to the distal pulmonary artery, involving multiple pulmonary artery segments. In addition, the higher incidence of syncope and the need for ICU may suggest a more severe clinical course. Furthermore, elevated cardiac markers, PAP, and more frequent right heart overload in these patients may indicate prominent early risk, consistent with previous studies [5, 8]. Furthermore, the higher incidence of syncope and the greater need for ICU in the presence of central thrombosis suggest that these cases have a more severe clinical course. However, the difference in platelet count was modest, with overlapping interquartile ranges, suggesting limited clinical relevance.

In line with these findings, studies support the use of quantitative scores reflecting thrombus burden in the risk assessment of central PE and suggest that these parameters may guide the determination of advanced treatment strategies [6, 14–16]. However, findings on clinical outcomes associated with thrombus localization are inconsistent and heterogeneous in the literature. It has been reported that the location of thrombus within the pulmonary artery tree is not always directly correlated with the clinical severity of PE [8]. The lack of a significant association between thrombus localization and inflammatory indices in our study may reflect the multifactorial nature of inflammation in APE. In addition to thrombus characteristics, factors such as hypoxia, comorbidities, and pulmonary parenchymal involvement may contribute to the systemic inflammatory response, potentially obscuring a direct relationship [1, 8].

Mechanical obstruction alone may be insufficient to explain the increased right ventricular afterload and associated right ventricular strain in PE [8]; it has been suggested that right ventricular dysfunction may also develop due to inflammatory processes. Indeed, in postmortem human studies and experimental models, cardiac tissue has been shown to be infiltrated with inflammatory cells [17, 18]. Furthermore, some studies have reported that more pronounced right ventricular dysfunction may develop in cases of peripheral PE [19]. This condition may be related to hypoxia, microvascular embolization, and the associated myocardial cell damage [4, 20]. On the other hand, some argue that the Qanadli score, commonly used in the quantitative assessment of thrombus burden, may not fully reflect the clinical severity of PE [6]. This may arise because a fixed score is assigned to each segment in the assessment of peripheral arteries, regardless of the degree of obstruction. In this context, alternative methods, including volumetric assessment of clot burden and integrated clinical–radiological scoring systems, have been proposed to provide a more comprehensive evaluation of disease severity than the Qanadli score alone [14].

Similarly, data on the role of the Qanadli score in predicting mortality also show heterogeneity. The general consensus is that mortality risk increases with right ventricular dysfunction in central PE with high thrombus burden [16]. Although it has been suggested that the degree of pulmonary artery obstruction alone may not be sufficient to determine prognosis [21]. In fact, numerous studies have failed to demonstrate a significant association between the obstruction index and mortality, and a high Qanadli score has been reported to have limited predictive power for mortality [7]. Similarly, in our study, high Qanadli scores and thrombus location were not significantly correlated with mortality, suggesting that anatomical assessment of thrombus burden alone may not be sufficient for predicting prognosis.

Our study has demonstrated that mortality is associated with increased systemic inflammation rather than thrombus burden or thrombus localization. A significant association between mortality and all evaluated inflammatory indices (SII, SIRI, and AISI) supports the importance of the inflammatory response in PE prognosis. In particular, the identification of SII and CRP levels as independent predictors of mortality in multivariate analyses suggests that these biomarkers may play a complementary role in clinical risk assessment. However, no significant relationship was found between inflammatory indices and thrombus load. This finding suggests that the inflammatory response in PE may not be solely related to thrombus formation, but rather reflects the involvement of more complex underlying mechanisms. Similarly, recent studies have examined biomarker-based composite scores, such as the albumin–bilirubin and platelet–albumin–bilirubin scores, which have shown prognostic value in APE, further underscoring the importance of integrated biomarker-based approaches to risk stratification [22].

The interaction between thrombosis and inflammation has long been studied under the concept of ‘thrombo-inflammation’, and it is well known that endothelial damage and hypercoagulability play important roles in the pathogenesis of PE [9]. Although thrombus burden and inflammation were evaluated as separate parameters in our study, these processes are biologically interconnected. Platelets and neutrophils play central roles in thrombosis and inflammation through mechanisms such as immunothrombosis and endothelial activation [9], suggesting an expected overlap between these pathways. However, the absence of a significant association between thrombus burden and inflammatory indices in our study indicates that systemic inflammation in APE may be influenced by factors beyond clot burden alone. Supporting this, studies in DVT have shown that although higher thrombus load and more proximal thrombi are associated with increased inflammatory indices, their discriminatory performance remains limited [23].

One possible reason for the lack of correlation between inflammatory indices and thrombus burden in our study may be that both the central and peripheral PE groups consisted of elderly patients. Age is known to independently affect systemic inflammation through comorbidities and chronic inflammatory conditions [24]. Furthermore, our study also demonstrated that age is an independent predictor of mortality in PE.

A possible reason for the inability to directly attribute inflammation to thrombus burden is the vascular remodeling in APE. During this process, pulmonary blood flow is directed toward better-perfused areas, and as a result, ground-glass opacities and consolidated areas may develop in the lung parenchyma. An analysis of CTPA findings reported that ground-glass opacities were detected in 48.4% of PE cases and parenchymal consolidations in 29% [1]. These findings suggest that a significant inflammatory process may accompany PE in the lung parenchyma, independent of thrombus presence.

Another mechanism that may affect the inflammatory response is the development of pleural effusion secondary to PE. In the literature, pleural effusion has been reported in approximately 35% of patients with PE [1], indicating that this finding is clinically significant. Pleural effusion is more frequent, particularly in cases of peripheral PE [1]. This may explain why no significant difference in inflammatory indices was observed between the central and peripheral embolism groups.

When these findings are considered together, it is clear that thrombus burden is not the only pathological mechanism contributing to systemic inflammation in PE. However, consistent with the literature, our study showed that mortality increases with increasing inflammation. Given the thrombogenic-inflammatory nature of PE, this result is expected. Similarly, in the study by Phan and colleagues, the neutrophil-to-lymphocyte ratio (NLR) was not associated with right ventricular dysfunction, troponin, or pro-BNP levels; however, high NLR values were significantly associated with all-cause mortality [25].

The PESI score has been widely used for many years in PE risk classification, but it was developed primarily from clinical parameters [4]. Therefore, the inability to adequately integrate imaging findings and hematological parameters may, to some extent, limit the prognostic accuracy of PESI. Early prognosis in acute PE is clinically important, as approximately 15% of hemodynamically stable patients still die from PE. Moreover, commonly used clinical risk stratification scores do not provide direct information regarding thrombus location or extent, nor do they adequately reflect the severity of the inflammatory response. In this context, the ESC risk stratification framework already incorporates the PESI score.

In our study, we found that SII, beyond being merely an inflammatory marker, may serve as a practical and accessible risk indicator for predicting poor prognosis in the early stages of APE. The AUC for SII in ROC analyses, along with high sensitivity and specificity, supports this interpretation. Although SIRI and AISI also demonstrate meaningful predictive performance for mortality, their lower discriminatory power compared to SII highlights the potential role of the platelet component in the prognostic process of PE. However, the prognostic value of SII should be interpreted alongside other inflammatory and biomarker-based indices, and more comprehensive comparative studies are needed to better define its clinical utility.

These findings are consistent with previous studies reporting that inflammatory indices are associated with PE severity and mortality. However, our study offers a unique contribution by evaluating the relationship among thrombus burden, localization, and inflammation. Thrombus localization is independent of inflammation severity, whereas inflammation plays a central role in determining prognosis. This supports the view that PE should be considered not only a mechanical obstruction disease but also a systemic inflammatory syndrome.

### Limitations

This study has some limitations. First, its retrospective, single-center design limits the generalizability of the findings and precludes causal inferences. In addition, inflammatory indices were assessed at a single time point on admission, which may not fully capture the dynamic nature of the inflammatory response. The Qanadli score used to assess thrombus burden may not fully reflect clinical severity because it is based on scoring methods for peripheral arteries. Furthermore, the fact that the CTPA images were evaluated by a single radiologist raises the possibility of observer bias and limits the reproducibility of imaging assessments. The study population, consisting of elderly patients with a high burden of comorbidities, may have affected systemic inflammatory levels independently of thrombus characteristics. Finally, the analyses were limited to 28-day mortality, and long-term clinical outcomes were not evaluated.

## Conclusion

This study demonstrated that, in patients with APE, thrombus burden and localization are associated with clinical severity, whereas mortality may be primarily linked to systemic inflammation. Although the Qanadli score was higher in centrally located emboli and appeared to be associated with hemodynamic effects, thrombus burden and localization alone were insufficient to predict mortality. In contrast, hematologic inflammatory indices, particularly SII and RDW, have emerged as independent predictors of mortality in acute PE. These findings support the notion that PE is not merely a mechanical vascular obstruction but also a significant thrombo-inflammatory process.

Peripheral blood cell count and CTPA are readily available and reproducible in clinical practice [26]. Our results suggest that adding low-cost, readily available inflammatory indices and imaging findings to current risk classification models may enable earlier and more accurate identification of high-risk patients, thereby offering clinical benefits. However, prospective, multicenter studies are needed to determine the role of these outcomes in clinical practice.

## Data Availability

The datasets used and/or analyzed during the current study are available from the corresponding author on reasonable request.

## Abbreviations

APE: Acute pulmonary embolism
PESI: Pulmonary embolism severity index
CTPA: Computed tomography pulmonary angiography
PE: Pulmonary embolism
SII: Systemic immune-inflammation index
SIRI: Systemic inflammatory response index
AISI: Aggregate index of systemic inflammation
ICU: Intensive care unit
HT: Hypertension
DM: Diabetes mellitus
COPD: Chronic obstructive pulmonary disease
AF: Atrial fibrillation
DVT: Deep vein thrombosis
SBP: Systolic blood pressure
DBP: Diastolic blood pressure
HR: Heart rate
RDW: Red cell distribution width
CRP: C-reactive protein
pro-BNP: B-type natriuretic peptide
SPO_2_: Peripheral oxygen saturation
PAP: Pulmonary artery pressure
ESC: European society of cardiology
SPSS: Statistical packages for the social sciences
ROC: Receiver operating characteristics
AUC: Area under curve
NLR: Neutrophil to lymphocyte ratio

## References

[1] Uzelac B, Jakovljević V, Živković V, Janković J, Lazarević K, Marković D, et al. Prognostic Value of Initial Inflammatory Biomarkers, ECG Findings, and Computed Tomography in the Assessment of Acute Pulmonary Embolism Severity. Medicina. 2025;61(10):1830. 41155817 10.3390/medicina61101830 PMC12565927

[2] Zhang Q, Abideen ZU, Shan KS, Yoon T, Farooq M. A silent fatal presentation of pulmonary embolism: reflection and discussion. Cureus. 2020;12(6). 10.7759/cureus.8813 PMC7384707 32742829

[3] Haba MȘC, Manole OM, Buburuz AM, Tudorancea I, Costache-Enache I-I, Onofrei V. The Prognostic Value of Inflammatory Indices in Acute Pulmonary Embolism. Diagnostics. 2025;15(3):312. 39941242 10.3390/diagnostics15030312 PMC11817101

[4] Zhang L, Ying K. A prognostic nomogram integrating CTPA with clinical and hematological parameters for pulmonary embolism. J Radiation Res Appl Sci. 2025;18(4):101935.

[5] Tiralongo F, Musmeci L, Tamburrini S, Sica G, Scaglione M, Tiralongo M, et al. D-Dimer/Fibrinogen Ratio and Radiological Severity Scores in Acute Pulmonary Embolism: Is There Room for a New Thrombus-Burden Marker? Diagnostics. 2025;15(22):2875. 41300899 10.3390/diagnostics15222875 PMC12651458

[6] Vamsidhar A, Rajasekhar D, Vanajakshamma V, Lakshmi A, Latheef K, Sankara CS, et al. Comparison of PESI, echocardiogram, CTPA, and NT-proBNP as risk stratification tools in patients with acute pulmonary embolism. Indian Heart J. 2017;69(1):68–74. 28228310 10.1016/j.ihj.2016.07.010 PMC5319130

[7] Vedovati MC, Germini F, Agnelli G, Becattini C. Prognostic role of embolic burden assessed at computed tomography angiography in patients with acute pulmonary embolism: systematic review and meta-analysis. J Thromb Haemost. 2013;11(12):2092–102. 24134450 10.1111/jth.12429

[8] Al-Dorzi HM, Almutawa FM, AlRuhaymi BA, Alhusaini AO, Alnamlah AM, Shaman AMB, et al. Characteristics, management and outcomes of central versus peripheral pulmonary embolism: a retrospective cohort study. Thromb J. 2025;23(1):22. 40087650 10.1186/s12959-025-00708-w PMC11908021

[9] Stark K, Massberg S. Interplay between inflammation and thrombosis in cardiovascular pathology. Nat Reviews Cardiol. 2021;18(9):666–82. 10.1038/s41569-021-00552-1 PMC8100938 33958774

[10] Gok M, Kurtul A. A novel marker for predicting severity of acute pulmonary embolism: systemic immune-inflammation index. Scandinavian Cardiovasc J. 2021;55(2):91–6. 10.1080/14017431.2020.1846774 33263444

[11] Özdemir L, Özdemir B, Gegin S, Aksu EA, Pazarlı AC. Can systemic inflammatory markers be used in pulmonary embolism risk assessment in patients with acute pulmonary thromboembolism? J Inflamm Res. 2025:5969–77. 10.2147/JIR.S514111 PMC12067453 40357377

[12] Furlan A, Aghayev A, Chang C-CH, Patil A, Jeon KN, Park B, et al. Short-term mortality in acute pulmonary embolism: clot burden and signs of right heart dysfunction at CT pulmonary angiography. Radiology. 2012;265(1):283–93. 22993221 10.1148/radiol.12110802 PMC3447174

[13] Riccio C. 2019 ESC Guidelines for the diagnosis and management of acute pulmonary embolism developed in collaboration with the European Respiratory Society (ERS). Eur Heart J. 2019. 10.1093/eurheartj/ehz405 31504429

[14] Huang W-M, Wu W-J, Yang S-H, Sung K-T, Hung T-C, Hung C-L, et al. Quantitative volumetric computed tomography embolic analysis, the Qanadli score, biomarkers, and clinical prognosis in patients with acute pulmonary embolism. Sci Rep. 2022;12(1):7620. 35538102 10.1038/s41598-022-11812-6 PMC9090848

[15] Senturk A, Ozsu S, Duru S, Cakır E, Ulaslı SS, Demirdogen E, et al. Prognostic importance of central thrombus in hemodynamically stable patients with pulmonary embolism. Cardiol J. 2017;24(5):508–14. 28248408 10.5603/CJ.a2017.0021

[16] Attia NM, Seifeldein GS, Hasan AA, Hasan A. Evaluation of acute pulmonary embolism by sixty-four slice multidetector CT angiography: Correlation between obstruction index, right ventricular dysfunction and clinical presentation. Egypt J Radiol Nuclear Med. 2015;46(1):25–32.

[17] Boyuk F. The role of the multi-inflammatory index as a novel inflammation‐related index in the differential diagnosis of massive and non‐massive pulmonary embolism. Int J Clin Pract. 2021;75(12):e14966. 34626044 10.1111/ijcp.14966

[18] Babes EE, Radu A-F, Babeş VV, Tunduc PI, Radu A, Bungau G, et al. The Prognostic Role of Hematological Markers in Acute Pulmonary Embolism: Enhancing Risk Stratification. Medicina. 2025;61(6):1095. 40572783 10.3390/medicina61061095 PMC12195037

[19] Kotasthane AN, Sridevi C, Malani SK, Jadhav A, Bhandari R. Comparative analysis of central and peripheral pulmonary embolism: Clinical profile, imaging findings, and outcomes in a tertiary care center. Heart India. 2025;13(3):231–7.

[20] Timurkaan M, Altuntas G, Kalayci M, Timurkaan ES, Ayyildiz H. Early warning triad for pulmonary microemboli in COVID-19 pneumonia: Pulmonary artery diameter, D-dimer and NT-proBNP. Medicine. 2022;11(2):775–9.

[21] Atik S, Ergün R, Ergün D, Narin Çopur E, Kılınçer A, Körez MK. The Role of the Pulmonary Artery Obstruction Index Ratio in Predicting the Clinical Course of Pulmonary Embolism. J Clin Med. 2025;14(5):1673. 40095678 10.3390/jcm14051673 PMC11900484

[22] Taş A, Tanık VO, Tunca Ç, Ateş MS, Oğuz F, Kılıç C, et al. Albumin–Bilirubin and Platelet–Albumin–Bilirubin scores as predictors of all-cause long-term mortality in intermediate–high-risk pulmonary embolism. Ir J Med Sci (1971-). 2026;195(1):105–16. 10.1007/s11845-025-04215-9 41348424

[23] Kuplay H, Erdoğan SB, Bastopcu M, Arslanhan G, Baykan DB, Orhan G. The neutrophil-lymphocyte ratio and the platelet-lymphocyte ratio correlate with thrombus burden in deep venous thrombosis. J Vascular Surgery: Venous Lymphatic Disorders. 2020;8(3):360–4. 10.1016/j.jvsv.2019.05.007 31405801

[24] Brinkley TE, Leng X, Miller ME, Kitzman DW, Pahor M, Berry MJ, et al. Chronic inflammation is associated with low physical function in older adults across multiple comorbidities. Journals Gerontol Ser A: Biomedical Sci Med Sci. 2009;64(4):455–61. 10.1093/gerona/gln038 PMC2657165 19196644

[25] Phan T, Brailovsky Y, Fareed J, Hoppensteadt D, Iqbal O, Darki A. Neutrophil-to-lymphocyte and platelet-to-lymphocyte ratios predict all-cause mortality in acute pulmonary embolism. Clin Appl Thromb Hemost. 2020;26:1076029619900549. 31960711 10.1177/1076029619900549 PMC7098206

[26] Shen C, Yu N, Wen L, Zhou S, Dong F, Liu M, et al. Risk stratification of acute pulmonary embolism based on the clot volume and right ventricular dysfunction on CT pulmonary angiography. Clin Respir J. 2019;13(11):674–82. 31344318 10.1111/crj.13064

